# Miscellany

**Published:** 1904-07

**Authors:** 


### Miscellany.

A More Simple Way of making a Crown.—A more simple way of making the crown described in the October issue, page 796, is as follows:

After the tooth is prepared, swage a gold cap to fit the case, reinforce the cusps with solder, cut out the buccal surface, and prepare as described. Then fuse the Jenkins body to this surface, and there you have it. This body can be baked before 18-carat, or even 14-carat, solder will flow.—N. A. Stanley.

---

New Application of Soft or Velum Rubber (Joaquin Plet, *La Odontologia,* Madrid, October, 1903).—In the class of cases where the two cuspids may be the only remaining teeth, with large crowns and narrow necks, the close adaptation of hard rubber in a denture around these teeth becomes impossible. The case is then flasked and the wax removed in the ordinary way. The plaster teeth may have been previously trimmed slightly, then completely encircled by packing liberally the soft rubber.

Around this is then packed the rubber ordinarily used, when the case is vulcanized as usual. Care should be observed in finishing not to use scrapers or files, but a sharp knife, with both the knife and rubber wet. Some little force will be required to press the plate to place, when a snug adaptation will be attained, offering support to the plate superior to clasps and less harmful to tooth-structure.—P. B. McC.

